# Quantitative methodology for poly (butylene adipate-co-terephthalate) (PBAT) microplastic detection in soil and compost

**DOI:** 10.1007/s11356-025-35978-4

**Published:** 2025-01-31

**Authors:** Yvan D. Hernandez-Charpak, Harshal J. Kansara, Jeffrey S. Lodge, Nathan C. Eddingsaas, Christopher L. Lewis, Thomas A. Trabold, Carlos A. Diaz

**Affiliations:** 1 Golisano Institute for Sustainability, Rochester Institute of Technology (RIT), Rochester, NY 14623 USA; 2 Thomas H. Gosnell School of Life Sciences, RIT, Rochester, NY 14623 USA; 3 College of Chemistry and Materials Science, RIT, Rochester, NY 14623 USA; 4 Department of Manufacturing and Mechanical Engineering Technology, RIT, Rochester, NY 14623 USA; 5 Department of Packaging and Graphic Media Science, RIT, Rochester, NY 14623 USA

**Keywords:** Microplastics, PBAT, GCMS, Biodegradation, Soil extraction, Microplastic detection

## Abstract

With the increasing use of biodegradable plastics in agriculture and food packaging, it has become increasingly important to assess the effects of their fragmentation and mineralization in the environment (i.e., soil, compost). PBAT is a biodegradable polyester widely used in biodegradable mulch films that are intended to fragment and mineralize in soil. To study these effects, novel methodologies are needed to quantify PBAT microplastics in these diverse environments. This work seeks to answer whether gas chromatography mass spectrometry (GCMS) can be used as a tool to assess PBAT microplastics in soil. A method was developed that allows PBAT soil extraction by ultrasonication and GCMS quantification after a fatty acid methyl ester derivatization. To validate the method, an industrial compost degradation experiment was carried out to evidence the weight loss of PBAT film and quantify the micro- and nano-plastic generated from them. The presented method improved the existing resolution by, at least, one order of magnitude compared to reported methods. In conclusion, a novel, simple, affordable, and reproducible methodology for PBAT microplastic detection was developed improving the limits of detection and quantification. The method was tested on an industrial compost experiment, demonstrating the ability to trace the totality of the plastic over time, evidencing that PBAT is consumed in the industrial compost environment.

**Supplementary Information:**

The online version contains supplementary material available at 10.1007/s11356-025-35978-4.

## Introduction

Growing populations and economic development require the use of agricultural mulching films (AMFs) for some crops, as they are inexpensive, easy to use, increase crop yield, diminish the use of pesticides and herbicides, and increase food quality (Espí et al. 2006; Gutierrez 2019; Kasirajan & Ngouajio 2012). The major setback is the disposal of the films at end-of-life, as it generates an additional cost to growers who must collect the films after each harvest and send it to landfills or incineration facilities, thereby increasing the environmental impact (Gutierrez 2019). The conventional plastic used for AMFs is low-density polyethylene (LDPE), due to its low price and excellent mechanical performance. During end-of-life collection, small amounts of the plastic are left behind leading to microplastic (MP) production (size < 5 mm) (T. Jin et al. 2022; United Nations Environment Programme 2022; Uwamungu et al. 2022). Agricultural plastic mulch usage is over 4 million tons annually and continues to grow at 5.6% per year (Ren et al. 2021) and LDPE does not degrade under normal environmental conditions, thus leading to an accumulation of MPs, that can hinder soil health (United Nations Environment Programme 2022; Yu et al. 2021). Artificial intelligence tools are being investigated to help automate analysis workflows and potentially improve detection efficiency but analytical methods such as infrared spectroscopy, nuclear magnetic resonances, gas chromatography with mass spectrometry, and Raman spectroscopy remain the basis for identifying and quantifying microplastics (H. Jin et al. 2024). Poly (butylene adipate-co-terephthalate) (PBAT) is a biodegradable polymer that has attracted industry and academia’s attention due to its ductility and good processability (Bhagwat et al. 2020; Kyrikou & Briassoulis 2007; Lawson & Taber 2011; Nunes et al. 2020; Sintim et al. 2020; Tan et al. 2016). Additionally, due to its low glass transition temperature, it has a relatively high rate of soil degradability, making it a promising alternative to replace conventional plastics in AMF applications (Touchaleaume et al. 2016; Zumstein et al. 2018). Most biodegradable AMFs are PBAT-based films that are compounded with other biodegradable resins like polylactic acid and even raw starch (Sintim et al. 2020; Touchaleaume et al. 2016).

The effects of biodegradable, PBAT-based, AMFs on soil health have been described by Bandopadhyay and collaborators, as indirect (via microclimate modification) and direct (via incorporation in soil) (Bandopadhyay et al. 2018). The indirect effects are somewhat similar to the LDPE-based films; however, the direct effects of the MPs are different. Astner and collaborators reviewed the interactions of MPs coming from the increasing AMF industry with agricultural soil ecosystems (Astner et al. 2023). Conventional films’ MPs accumulate with time, are “easily” detectable and their lack of degradability leads to a continuous increase in their impact (Astner et al. 2023; He et al. 2018; T. Jin et al. 2022; Uwamungu et al. 2022; Zhang et al. 2018). Uwamungu and collaborators described their impacts on the different soil cycles such as bacterial, fungal, and plant growth cycles (Astner et al. 2023; Uwamungu et al. 2022). PBAT-based films degrade with time, leading to a stabilization of the MP concentration (Yu et al. 2021). Even though their impact is less than MPs coming from conventional films, it is important to monitor and study how it affects microbial, fungi, and plant cycles (Astner et al. 2023; Bandopadhyay et al. 2018). Nie and collaborators found negative impacts of 0.5% of PBAT MPs on bacterial abundance and diversities in water columns (Nie et al. 2022). On the other hand, Li and collaborators found that low content of PBAT MPs (0.02%) showed slight increase in diversity of landfill bacterial communities, but high contents (> 2%) would result in a decrease in bacterial diversity, concluding the existence of a certain dose–effect for different amounts of PBAT (Li et al., 2022a). Liu and colleagues found similar results on latosol microbial diversity and dissolved organic matter, since low concentrations of PBAT (5%) showed an increase in dissolved organic matter, however, high concentration (10%) diminished the microbial diversity. Additionally, the authors reported that PBAT MPs impact on fungal richness is different than that for microbial activity (Y. Liu et al. 2023). The impact of PBAT microplastics on environment is influenced by their concentration, and due to its biodegradability, this concentration is not constant. Astner and coauthors confirm that additional long-term studies are required (Astner et al. 2023), highlighting the need to assess PBAT MP concentration in and out of the laboratory setting to continue to study their effect on agricultural soils.

The quantification of PBAT from different environments is a challenge. Cho and collaborators developed an effective gas chromatography coupled with mass spectrometry (GC–MS) method to quantify degraded PBAT film in wastewater (Cho, Kim, et al., 2022; Cho, Park, et al. 2022a, b). The method dissolves the PBAT in chloroform (CHCl_3_) and performs a fatty acid methyl ester derivatization (FAME) to break down the polymer chains allowing quantification of PBAT in CHCl_3_ (Cho, Kim, et al., 2022). However, the limit of detection (LOD) and of quantification (LOQ) were reported to be 0.26 g/L (260 ppm) and 0.80 g/L (800 ppm), respectively, which are uncommonly high for a GCMS methodology (Wollein & Schramek 2012). The potential of a methodology using FAME and GC was also detailed by Wortman and collaborators (Wortman et al. 2022). They reported a clear detection from soil of PBAT-based plastics; however, they did not assess concentration of PBAT in the soil. The lowest detected amount they reported was 0.08 mg of PBAT-based films [26]. Nelson and collaborators developed an analytical methodology to quantify microplastics from PBAT in soils (Nelson et al. 2019). Using proton nuclear magnetic resonance (^1^H-NMR), they extracted PBAT in CHCl_3_ and compared it with 1,4 dimethoxybenzene in deuterated-CHCl_3_ (CDCl_3_), allowing them to quantify very effectively the PBAT concentration. Their LOD and LOQ were determined to be 1.3 and 4.4 µg/mL of CDCl_3_, respectively. However, this methodology uses deuterated chloroform for the analysis, which is an expensive solvent, and their LOD and LOQ depend on the PBAT extraction from soil (Nelson et al. 2019). The authors explore three different extraction methods in the laboratory: Soxhlet, accelerated solvent, and ultrasonication. Briefly, the accelerated solvent outperforms the others but demands a specific (and expensive) instrument; the Soxhlet extraction is usually used for non-soluble and is more expensive. Finally, ultrasonication is the most accessible method but more labor intensive; however, it performs similarly to the two other methods (Nelson et al. 2019). In a review, Astner details the limits of NMR for PBAT quantifications due to the solvent-soil organic matter interactions leading to interferences in the spectra (Astner et al. 2023). This, however, should be less of a barrier using GCMS, which is a widely used methodology to find components in highly contaminated soil samples (Cho, Park, et al. 2022a, b; Profumo et al. 2020). In the presented work, we therefore detailed and tested in a real-world application, an accessible and reliable methodology that combines the ultrasonication process of the NMR methodologies (Astner et al. 2023; Nelson et al. 2019) with the FAME and GCMS analysis presented by Cho and collaborators (Cho, Kim, et al., 2022; Cho, Park, et al. 2022a, b).

## Methodology

### Materials

PBAT (EcoWorld®) was supplied by Jinhui Zhaolong High Technology Co. (Shanxi Province, P.R. China) with a melt flow index of $4.8\pm 0.4$ g/10 min (190 °C, 2.16 kg, by ASTM D1238-A). Using this material, PBAT films for composting were manufactured in a three-layer blown film line Labtech Engineering Co. Thailand (model LF-400-COEX). The equipment utilizes three single-screw extruders feeding into a co-extrusion annular die with a 50 mm outside diameter and a gap of 0.8 mm. The extruders have a barrel diameter of 20.0 mm with an L/D = 30. For this work, only one extruder was used at 15 rpm, with temperatures of 180 °C, 180 °C, 175 °C, 170 °C, and 160 °C from feed to the die to achieve a melting temperature of 170 °C. The blow-up ratio (BUR) was maintained between 1.8 and 2.5 so the film thickness was kept under 75 µm; this led to a draw-down ratio (DDR) between 3.5 and 6. The ratio between rollers was maintained between 2 and 3 to avoid slipping.

The soil used in the work corresponds to a garden amendment (Bovung manure blend, Scotts Miracle-Gro Company, Marysville, OH, USA). It is a store-bought soil to allow replicability and consistency of the work (Bhattacharya et al. 2023; Hernandez-Charpak et al. 2024).

The calibration curves detailed in the following section were made with cryoground PBAT pellets as suggested by literature (Astner et al. 2019; Hrovat et al. 2024; Seghers et al., n.d.; Tewari et al. 2022). Briefly, pellets were pulverized in a SPEX SamplePrep 6870 Freezer/Mill® (SPEX SamplePrep, Metuchen, NJ, USA) High-Capacity Cryogenic Grinder (“cryo-mill”). The cryo-mill is a cryogenic grinder that contains one dual chamber, in which samples are continuously submerged in liquid nitrogen to maintain cryogenic temperatures. Approximately 30–35 g of pellets was placed in the cryo-mill, and each sample underwent four cycles with a 10-min precool, a 6-min cycle time, and a 3-min cooling in between cycles, with a rotation rate of 15 rpms. The particle size of the resulting powder was measured by stereo-microscope inspection to be less than 100 µm, which agrees with literature using similar methods (Astner et al. 2019; Hrovat et al. 2024; Seghers et al., n.d.; Tewari et al. 2022).

### Chemicals

Two different grades of chloroform (CHCl_3_) were used in this work, chloroform 1 (Sigma-Aldrich, C2432, 100–200 ppm amylenes as stabilizer, ≥ 99.5%) and chloroform 2 (Macron and VWR Chemicals, ACS grade). Sulfuric acid (Barker, ACS grade), methanol (VWR, ACS grade), calcium chloride anhydrous (Sigma Aldrich, free-flowing, ACS reagent ≥ 96%), and water (EMD Corporation, HPLC grade) were also used.

### Sample preparation

#### Soil extraction

To extract microplastics, an ultrasonication method was used following (Nelson et al. 2019; Profumo et al. 2020). Chloroform was chosen due to its versatility as a solvent for biodegradable polymers; the corresponding safety precautions must be taken as it is a dangerous chemical. Handling and sample preparation must happen under chemical hood and protecting skin. Multiple iterations on time and temperature led to the final protocol as follows: First, mix 10 g of dried soil (24 h at 105 °C) with 30 mL of CHCl_3_, sonicate (AUCMA, China, 43 kHz) for 35 min at 60 °C. Once finished, allow 15 min of cool down in the fridge to bring it to room temperature. Then, filtrate the solution through a Whatman paper filter 40 and wash with 30 mL of additional CHCl_3_. Follow with an additional filtration through a glass syringe with a Whatman filter PTFE 0.45 µm into a 100 mL round bottom flask. Next, solvent was removed using rotary evaporation (60 rpm/35 °C). Finally, redissolve the remains of the flask in 2 mL of CHCL_3_. This solution can be stored in the fridge for further analysis. Additional description of the iterations that obtained this protocol can be found in the supplemental information.

#### Fatty acid methyl ester derivatization (FAME)

To depolymerize the PBAT microplastics in soil, a FAME method following (Cho, Kim, et al., 2022; Cho, Park, et al. 2022a, b) was used. Briefly, it starts with diluting 10 µL of sample from the final solution of the soil extraction in a 10 mL volumetric flask with CHCL_3_. Further, dilute by transferring 200 µL in 1.8 mL of CHCl_3_. Follow by adding 2 mL (1:1) of methanol/sulfuric acid (85:15 v/v). Then, heat at 105 °C for 2 h on a heating plate. Reinforcing the seal with teflon tape is advised to avoid loss of the CHCl_3_. Once finished, allow 15 min of cool down in the fridge to bring the solution to room temperature. Continue by adding 2 mL (1:2) of H_2_O and vortex gently for 1 min. Follow by separating the organic phase layer (bottom) in a glass essay tube containing anhydrous calcium chloride to completely remove the water. Finally, filter (Nylon, Fisherbrand, pore size, 0.2 µm or 6900–2502 GD/X PVDF, Whatman, pore size, 0.2 µm) the solution into a GCMS analysis vial.

The initial dilutions of the FAME procedure are needed to lower the concentration to the levels of the instrument. If the samples are expected to have a concentration of ~ 1 mg/mL of CHCl_3_ (1 mg/g of dry soil), these steps will dilute it to ~ 0.1 µg/mL of CHCl_3_, which is an adequate concentration for detection for the instrument. The control stock solution was made by dissolving 10.0 mg of cryo-grounded PBAT in 10.0 mL of CHCl_3_ (~ 1 mg/mL of CHCl_3_ or 1 mg/g of dry soil). From this stock solution, the samples for the calibration curve were made with the FAME proceeding but with different dilutions. Table 1 details the concentrations of the calibration samples, different than the second dilution (e.g., 200 µL in 1.8 mL) of the above protocol, to obtain nominal concentrations.

**Table 1:** Calibration curve sample concentrations

Nominal concentration (ng/mL)	1 µg/mL solution (mL)	Pure CHCl_3_ (mL)	Type of CHCl_3_
50	0.1	1.9	1 and 2
100	0.2	1.8	1 and 2
150	0.3	1.7	1 and 2
200	0.4	1.6	1 and 2
250	0.5	1.5	1 and 2
500^*^	1.0	1.0	2 only
750^*^	1.5	0.5	2 only

^*^Only for the second calibration curve

With each set of samples, an additional control was made to determine a response factor correction so the measured response could be compared to the calibration curve.

### Gas chromatography

The samples were analyzed using GC–MS (Shimadzu, GCMS-QP2020nci) equipped with a fused silica capillary column (RXI-1MS, Restek, 30 m × 0.25 mm i.d. × 0.25 µm). The GC method consisted of a starting temperature in the oven of 50 °C for 1 min, followed by a linear temperature gradient at a rate of 15 °C/min to 120 °C. The temperature was held for 2 min at 120 °C, and then increased at a rate of 10 °C/min to 300 °C, which was held for 5 min. The injector port temperature was set to 250 °C, and the injection mode was split with a ratio of 50, using helium as carrier gas with a column flow of 1.05 mL/min and a purge flow of 3.0 mL/min. The injection volume was set to 3 µL.

### Mass spectroscopy

To protect the detector from the multitude of volatiles and semi-volatiles present in the soil, the MS was set in selected ion monitoring mode (SIM) and turned on only during two-time windows. Based on the results of Cho and collaborators, PBAT samples would only yield two peaks of interest from the GC. The first at around 8.6 min, and a second at 12.7 min [24]. Therefore, the MS detector was turned on from minutes 8 to 10 and from minutes 12.25 to 14. The ions to monitor were chosen from the spectra presented in the work of Cho et al. [24] and confirmed by a control sample from the calibration curve (see “Results and discussion” Section) that ran in total ion count (TIC) mode. For the adipic acid, dimethyl ester peak (RT: 8.6 min) the ions 114, 74, and 59 m/z were followed. For the terephthalic acid, dimethyl ester peak (RT: 12.6 min) the ions followed were 163, 194, 135, and 59 m/z. The MS electron impact ionization was configured at 70 eV. The GCMS analysis is depicted in Fig. S1 of the Supplemental Material.

### Soil extraction experiment

To assess the extraction efficiency of this methodology, samples of 0.1, 0.15, 0.2, 0.25, 1.0, 1.5, 2.0, and 2.5 µg of PBAT diluted in CHCl_3_ (from a 10 mL solution of concentration 1.0 µg/mL) were each deposited in 10 g of dry soil. The same nominal concentration samples were made in CHCl_3_ from the same stock solution. The comparison of the resulting calibration curves allows us to assess the quality of the method to quantify PBAT in soil.

### Industrial compost experiment

The industrial compost was carried out over 90 days, with sampling at days 14, 23, 50, 59, 66, 73, 80, and 90. The temperature of the samples and the compost are shown in Fig. S2. The aerated static pile (ASP) was ~ 4 m tall and ~ 8 m wide and structured as follows: 3.75 m of a mixture of food waste and 25 cm cover of woodchips; the experiment was conducted at a large-scale industrial composting facility in Henrietta, NY. From the PBAT film described above, 75 mm-side squared samples were cut, in triplicates, and inserted in 1-mm holes PVC mesh bags (Saint-Gobain ADFORS) along with 100 g of sieved soil (< 5 mm) to attempt to maximize the uniform contact between the film and the compost environment. The composting process was conducted under controlled conditions, with temperatures maintained above 60 °C for most part throughout the duration of the experiment. This temperature range is characteristic of the thermophilic phase of composting, which is critical for the degradation of biodegradable polymers like PBAT due to heightened microbial activity and accelerated hydrolysis of ester bonds. A more detailed description is found in the supplemental information, specifically Figs. S3 and S4.

So as not to lose the sample bags into the large pile, bags were placed in eight identical 5-gallon bins. Each bin represented one time point for sampling. Bins were then filled with composting material and the mesh bags placed into the bins about 2 to 3 cm apart from each other. After which the bins were placed in the center of the pile (~ 1 m from the ground) and covered with about 2–2.5 m of composting material. Mesh bags were carefully retrieved from the bins and stored in Ziplock bags to be transported back to the lab. Mesh bags were opened over a clean aluminum foil and the contents pulled out and dried at 60 °C for 24 h. The soil was then sieved through a size 14 mesh (1.4 mm); plastic pieces over the mesh were retrieved and weighed. The soil was kept and used with this methodology to quantify the PBAT micro- and nano-plastics attached to it.

PBAT film weight loss was assessed as shown in Eq. 1:

1 $$W{L}_{n}\left[\%\right]=\left(1-\frac{{W}_{n}}{{W}_{0}} \right)\times 100\left[\%\right]$$

where ${W}_{n}$ is the composted and cleaned sample weight and ${W}_{0}$ is the initial sample weight. The standard deviation of the weight loss was propagated from the standard deviations of the weight averages, following Eq. 2:

2 $${\sigma }_{W{L}_{n}}=\frac{{W}_{n}}{{W}_{0}}\sqrt{{\left(\frac{{\sigma }_{0}}{{W}_{0}}\right)}^{2}+{\left(\frac{{\sigma }_{n}}{{W}_{n}}\right)}^{2}-\frac{2*{\sigma }_{n0}}{{W}_{n}*{W}_{0}}}$$

where ${\sigma }_{i}$ is the standard deviation of the measurement $i$ and ${\sigma }_{n0}$ is the covariance of measurements 0 and $n$.

The consistent high temperature of the composting environment provided ideal conditions for enzymatic activity, fragmentation, and subsequent microbial assimilation of PBAT. Sampling intervals were chosen based on the typical compost pile turning times of the facility to ensure minimal interruption to the composting process and the pile mature times established by the facility. The brittle and thin nature of PBAT films after composting can exacerbate fragmentation during retrieval, potentially contributing to an overestimation of microplastics. To address this, careful handling protocols were implemented during the retrieval process, and fragments generated during handling were analyzed alongside those from the screening process. While the integration of GC–MS analysis minimized potential underestimation of microplastics, future studies should explore improved retrieval techniques and sieving methods to reduce errors associated with the brittle nature of composted films.

## Results and Discussion

### Calibration curves

**Fig. 1:**
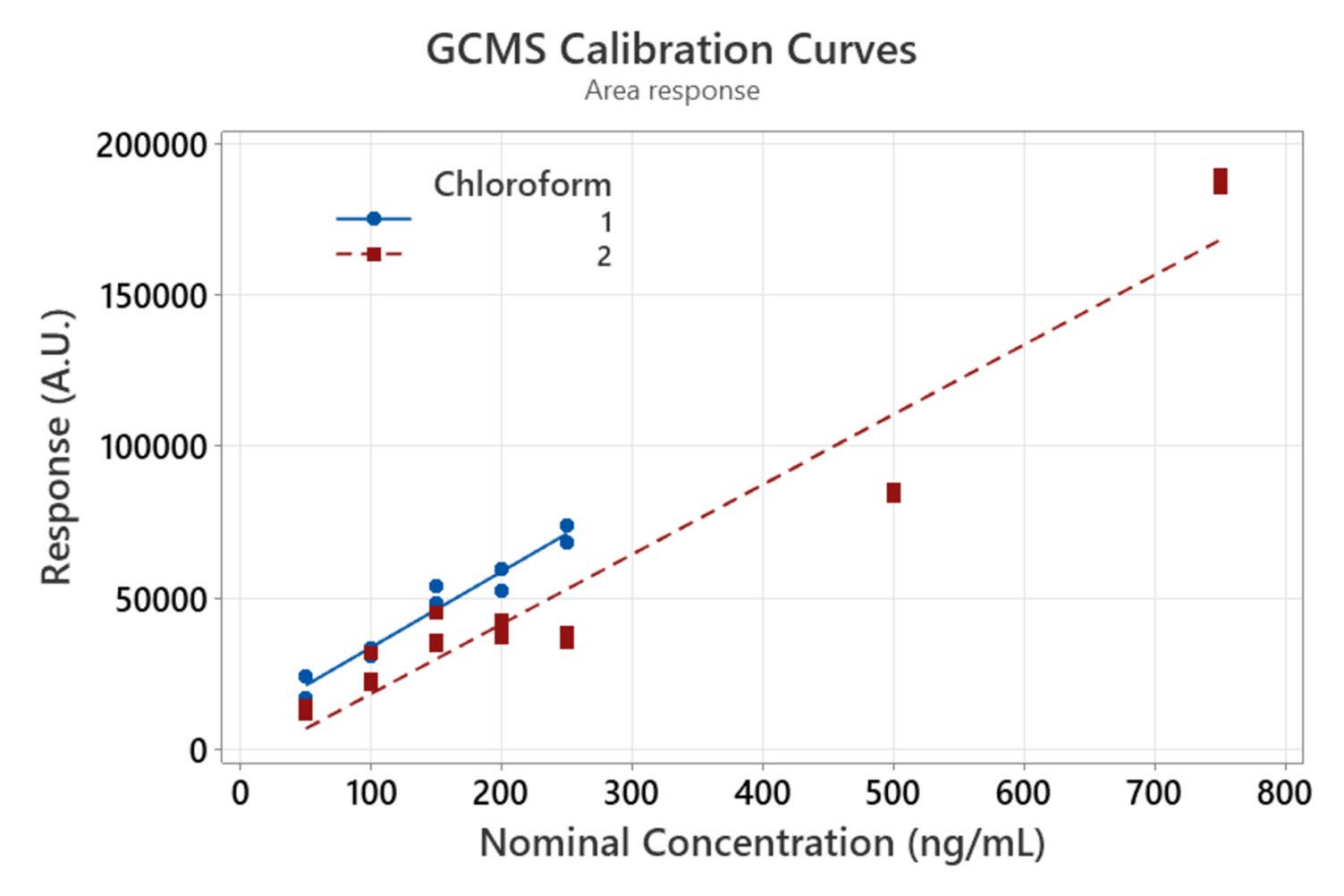
Calibration curves relating GCMS response with adipic acid dimethyl ester concentration in CHCl_3_

The first calibration curve was made with five samples at 50, 100, 150, 200, and 250 ng/mL of CHCl_3_ concentrations, as shown in Table 1. The compound used to quantify the co-polymer was adipic acid dimethyl ester. Figure S1 in the supplemental information details the process of quantifying the GCMS response and the rules for consistency in the analysis, i.e., manual integration for the area response of the ionic response. The second calibration curve was made with two additional samples (with concentrations of 500 and 750 ng/mL). Figure 1 shows the linear fits of each calibration curve. The first resulted in an equation of $y=8786+250.2x$ with ${R}^{2}=0.951$ and the second calibration curve was $y=-4784+230.4x$ with an ${R}^{2}=0.929$.

**Fig. 2:**
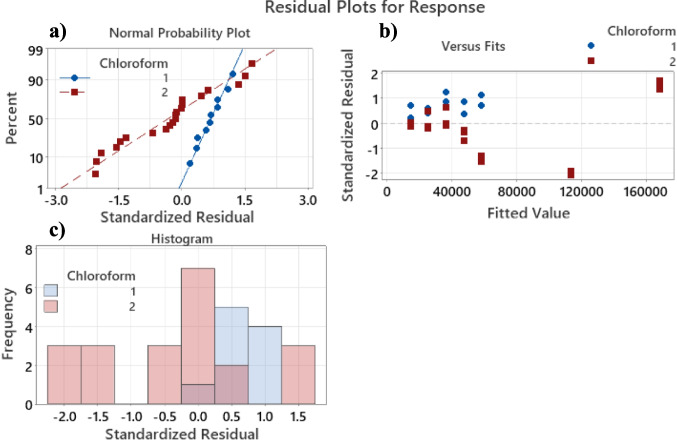
Residual analysis of the linear regressions, evidencing the absence of outliers and normality of the standardized residuals

The 95% confidence interval of the slopes are $\left[204.0;296.4\right]$ and $[199.7;261.1]$ for the first and second calibration curves, respectively. The overlap of the intervals indicates that slopes are equivalent, independent of the used chloroform. Figure 2 shows the residual analysis of the regressions. Figure 2a shows the normal probability plot of the residual from the regression of Fig. 1; these residuals appear to be normal with a *p* value of 0.07 (93% of confidence). Figure 2b shows the larger values of the calibration curve are within reasonable error, even though they are close to being outliers (less than a standardized residual of 2). Figure 2c tells a similar story than Fig. 2a, suggesting a normal shape of the residual distribution; however, an interesting detail is the positive sign of the residuals of the chloroform 1, explaining the high *p* value of the normality test.

### Soil extraction experiment

**Fig. 3:**
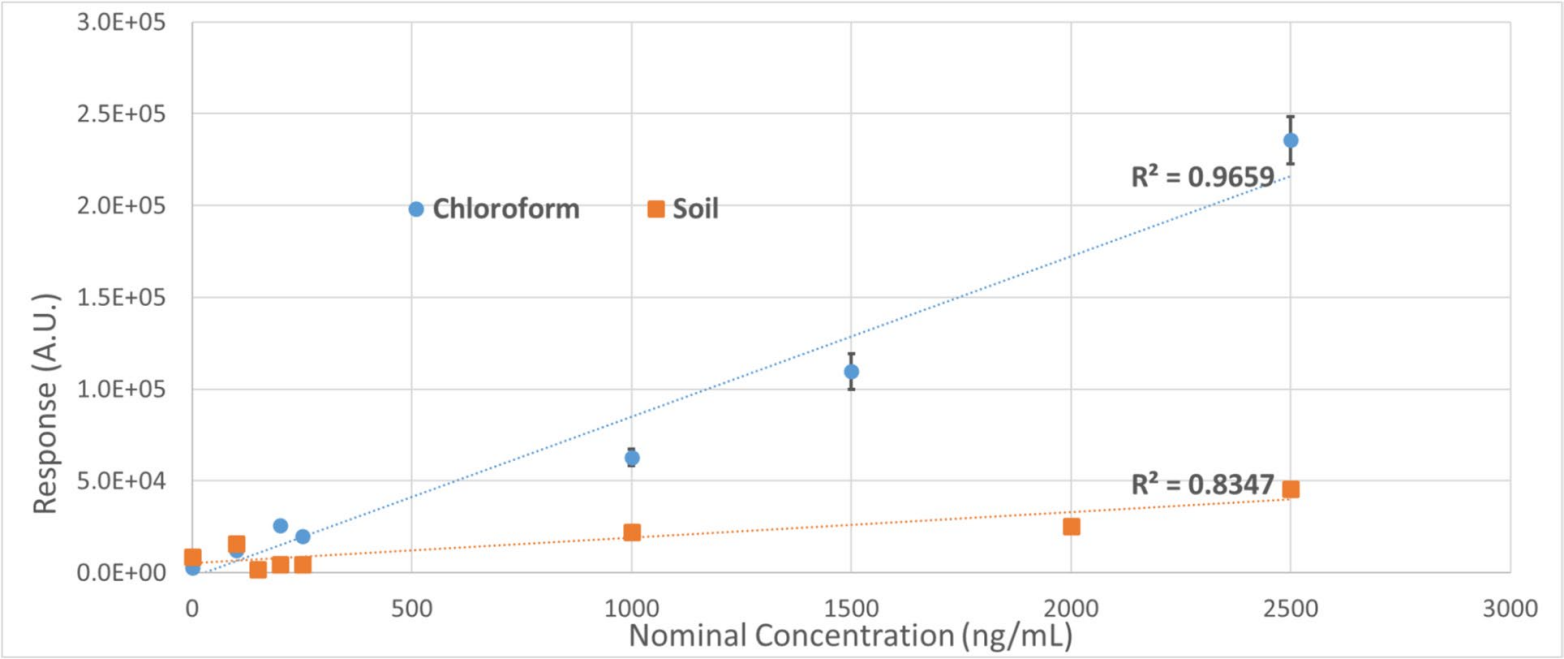
Soil extraction experiment result. Detection of adipic acid dimethyl ester from chloroform versus soil. The detection and quantification limits from soil are shown to be about 5 times less sensitive than from chloroform

Figure 3 displays the results from the calibration curve from soil extraction compared to the one from chloroform. The sensitivity of the method diminishes due to the heterogeneity of the soil, because the extraction process can dissolve a wide variety of compounds than will hinder the ability to detect the adipate acid.

Table 2 shows the summary of the theoretical limits of this work compared with literature. To calculate the theoretical LOD and LOQ of the presented method, Eqs. 3 and 4 were used:

3 $$LOD=3.3\times \frac{{\sigma }_{m}}{m}$$

4 $$LOQ=10\times \frac{{\sigma }_{m}}{m}$$

where ${\sigma }_{m}$ is the average of the standard deviations of the triplicate measurements used in the calibration curves and $m$ the slope of the calibration curve.

**Table 2:** Limits of detection and limits of quantification of PBAT concentration

Method	LOD	LOQ	*R*^2^
GCMS from CHCl_3_ (from the three independent calibration curves of this work)	0.102 ± 0.053 µg/mL	0.34 ± 0.178 µg/mL	0.95 ± 0.02
GCMS from CHCl_3_ (Cho, Kim, et al., 2022; Cho, Park, et al. 2022a, b)	260 µg/mL	800 µg/mL	0.996
GCMS from soil (this work)	0.44 µg/mL	1.5 µg/mL	0.83
NMR from soil (Nelson et al. 2019)	1.3 µg/mL	4.4 µg/mL	1.00

The GCMS’s LOD and LOQ from CHCl_3_ are consistent with traditional GCMS detection levels, almost 3 levels of magnitude lower than the found literature, probably due to an instrumentation problem. As discussed in the protocol, the single ion monitoring is key to not saturate the detector. We lowered LOD and LOQ compared to the NMR methodology of Nelson and collaborators making GCMS a relevant method for biodegradable microplastic analysis. However, the method presents a lower *R*^2^ that can be explained by the large number of calibration levels lower than the calculated LOQ. It does, however, show the relevance of these samples, as they are mostly explained by linear regression.

### PBAT microplastics in industrial compost

**Fig. 4:**
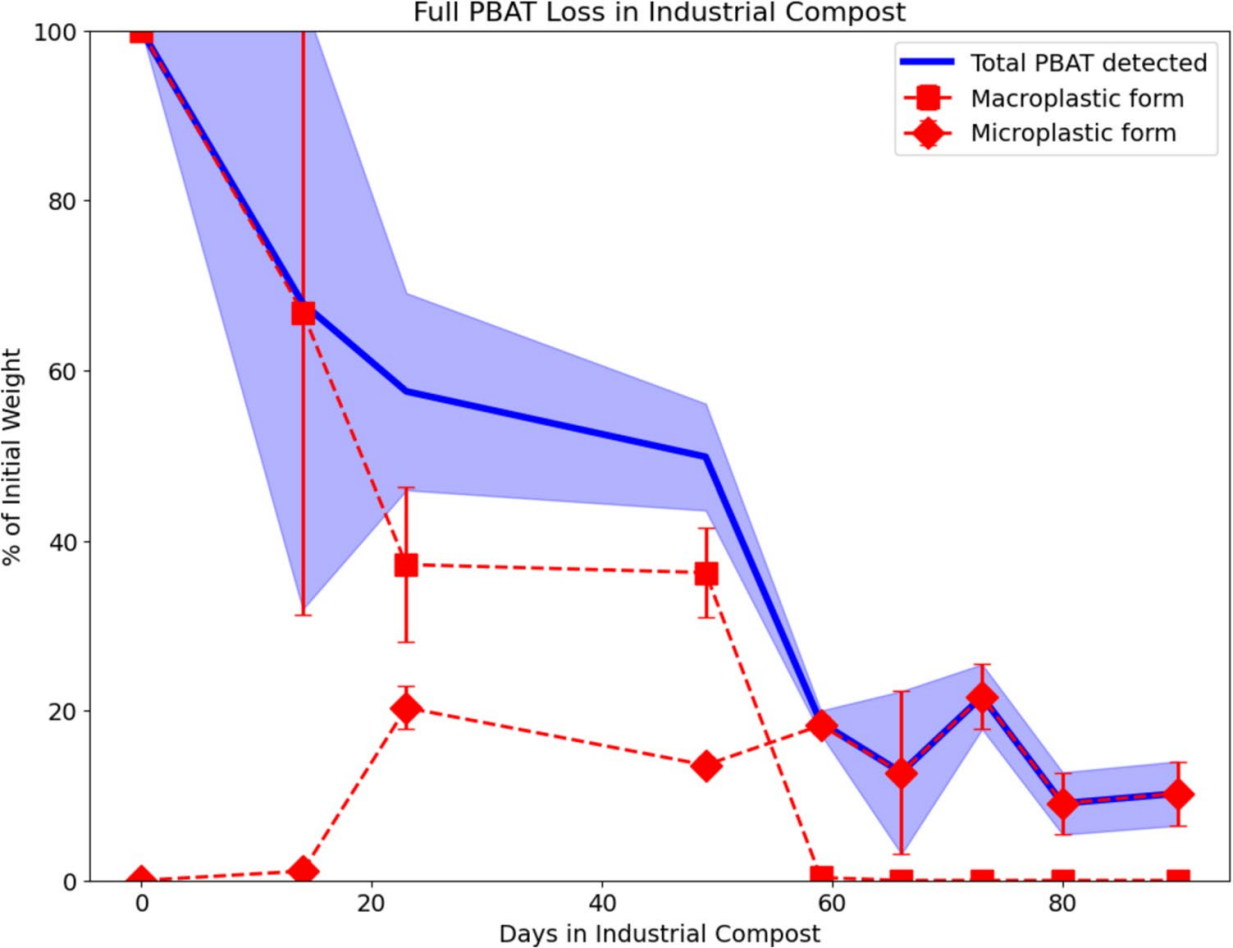
Full PBAT mass loss obtained by combining the data from the film weight loss and the microplastic concentration detected with GCMS in the surrounding soil of the mesh bag. The shaded region corresponds to the standard deviation of the total accounting of the film weight; this is the combination of both micro- and macro-plastic detection

Figure 4 depicts the evolution of the total amounts of PBAT, a combination of macro- and microplastics, during the course of degradation in compost. By knowing the initial amounts of PBAT inserted in the mesh bag and the amount of soil surrounding the samples, this method helps estimate the fraction of PBAT that corresponds to micro- and nano-plastics. The range of total PBAT detected moves away from the initial amount after 23 days, suggesting monomer consumption as the GCMS cannot account for the amount corresponding to total adipic acid. Even though the film was fully fragmented after 59 days, the micro- and nano-plastics persist 30 days after, representing close to 10% of the initial weight at the end of the experiment.

This work presents an accessible methodology to accurately detect small amounts of PBAT and follow their evolution, including in complex and heterogeneous environments like soil or compost. Based on this data, it is not unreasonable to think that after 120 days of industrial compost, no remaining PBAT film residue will be detectable. Additional to the detection potential for PBAT presented here, this method should be easily reproduceable for other biodegradable polyesters also mentioned in the referenced work such as polycaprolactone (PCL), polyhydroxy butyrate (PHB), and polybutylene succinate (PBS) (Cho, Kim, et al., 2022). This method can additionally be used to enhance the understanding of the effects of PBAT microplastics on soil and plant health (Li et al., 2022b; L. Liu et al. 2022; Muroi et al. 2016) and follow more closely microplastic production from mulch films in field trials (Ghimire, Scheenstra, et al., 2020; Sintim et al. 2020).

It is important to understand the limitations of this methodology as well. Soil type and composition can lead to changes on extraction efficiency (Nelson et al. 2019). Table 3 shows the soil analysis of the final material in the bins. A fairly neutral soil with high organic matter shows a healthy compost helping this analysis but more complex matrices could lead to a reduction of the effectiveness of the method.

**Table 3:** Soil analysis of organic matter retrieved at the end of the industrial composing

Organic matter (%)	39.20%
pH	7.55

Soil microplastics travel, migrate, and interact with different actors within soil (T. Jin et al. 2022); hence, this methodology should be applied in concert with a rigorous sampling methodology such as soil quartering (Ghimire, Flury, et al., 2020). Additional experimentation is needed to tackle the reported variability for different soil types, and that need of specific solvent mixes for the specific biodegradable polymers (Nelson et al. 2019). This method shows promising LOQ and LOD soil concentrations but the sensitivity and variability within instrumentation also needs to be investigated, GCMS can have dramatically different sensitivities based on their maintenance and protocols (Cho, Kim, et al., 2022; Wollein & Schramek 2012). However, this method is an effective step in addressing the need for additional and versatile ways to detect biodegradable polymers microplastic in soils.

## Conclusions

In this study, gas chromatography-mass spectrometry (GCMS), a widely used and available technique with low-cost solvents and chemicals, was utilized for detection of poly (butylene adipate-co-terephthalate) (PBAT) microplastic detection in soil and compost. The methodology presented here, although with a chemical pre-treatment (FAME), shows the potential for better resolution than the available methods in literature. The work presented evidence for only store-bought soil but proved to work in complex environments such as industrial compost. This sets up the stage for additional work and method optimization so it can be used on agricultural soils that utilize PBAT-based mulch films or other biodegradable polyesters for which the FAME would be needed. The combination of these factors makes this protocol a simple, affordable, and reproduceable method for the detection of PBAT microplastic in soil and compost.

Supplementary information.

## Supplementary Information

Below is the link to the electronic supplementary material.

ESM 1 (DOCX 5.94 MB)

## Data Availability

The authors declare that the data supporting the findings of this study are available within the paper. Should any raw data files be needed in another format they are available from the corresponding author upon reasonable request.
