# Supplementary material for: Quantitative methodology for poly (butylene adipate-co-terephthalate) (PBAT) microplastic detection in soil and compost
Source: Environ Sci Pollut Res Int. 2025 Jan 31;33(6):1755–64. doi: 10.1007/s11356-025-35978-4 (PMC12960377; doi:10.1007/s11356-025-35978-4)
Supplement: Supplementary file 1 — (DOCX 5.94 MB) [file 11356_2025_35978_MOESM1_ESM.docx]

**Quantitative methodology for poly (butylene adipate-co-terephthalate) (PBAT)**

**microplastic detection in soil**

Yvan D. Hernandez-Charpak^1^, Harshal J. Kansara^1^, Jeffrey S Lodge^2^, Nathan C. Eddingsaas^3^, Christopher L. Lewis^4^, Thomas A Trabold^1^, Carlos A. Diaz^5^

*Iterations on the soil extraction method*

The sonication time was explored between 24 minutes to 48 minutes (12 minutes increment) the effectivity of detection on the two last times (35 and 48) was found equivalent. The sonication temperature was given by the instrument, a trial with additional heat was attempted but the pressure of the heated CHCl_3_ broke the seal of the vessel.

*Iterations on derivatization*

Changing the final filters for PTFE filters (same pore size) was attempted unsuccessfully. For some reason, it did not allow the detection of monomers.

Figure S1. Process of quantification of GCMS response of an example sample of 150 ppb.


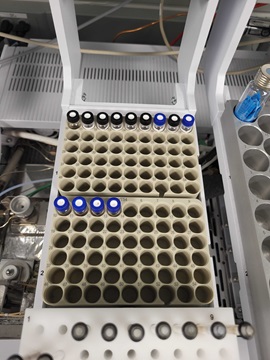

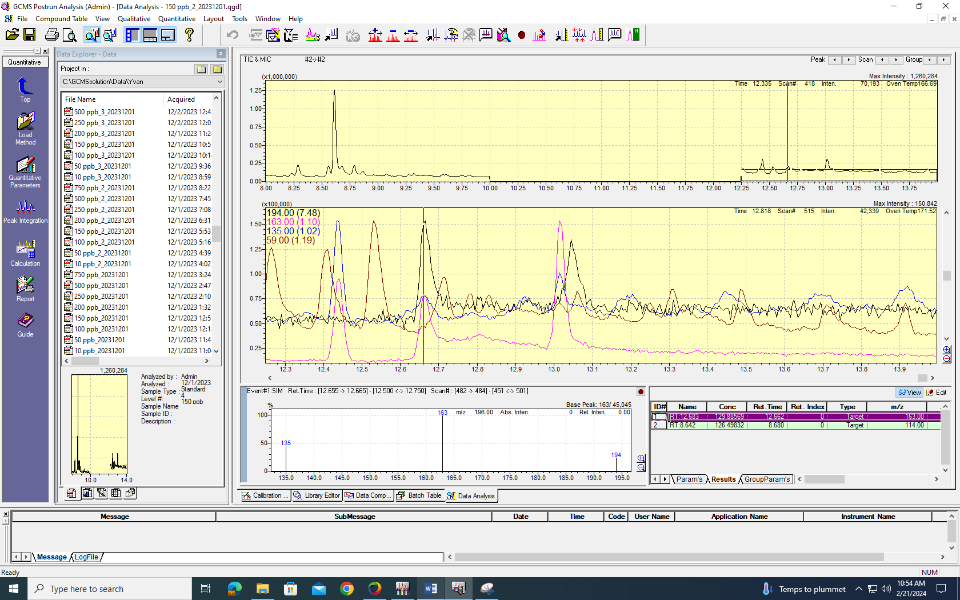

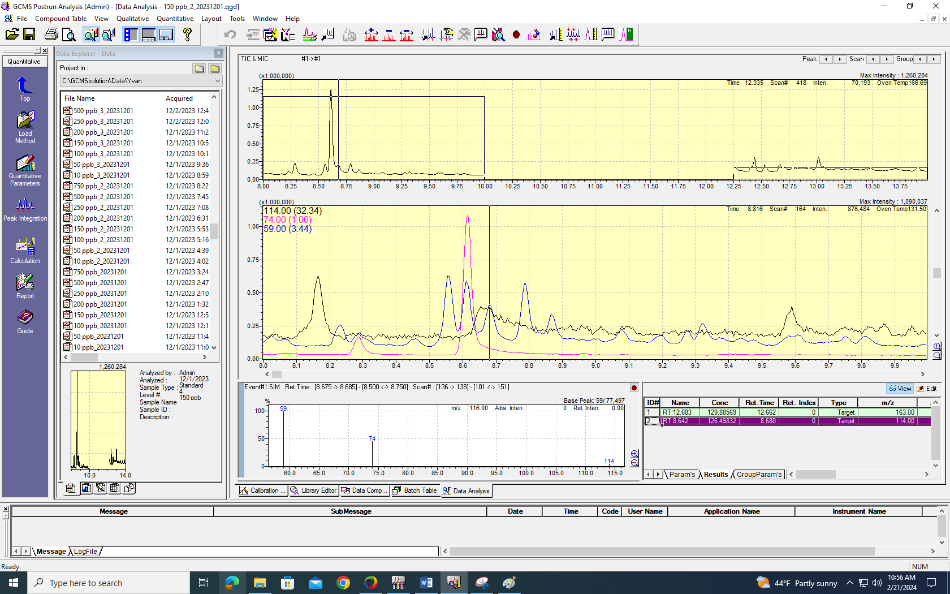


Selected Ionic Monitoring

Adipic Acid, dimethyl ester (RT: 8.6 min)

Terephthalic acid, dimethyl ester (RT: 12.6 min)


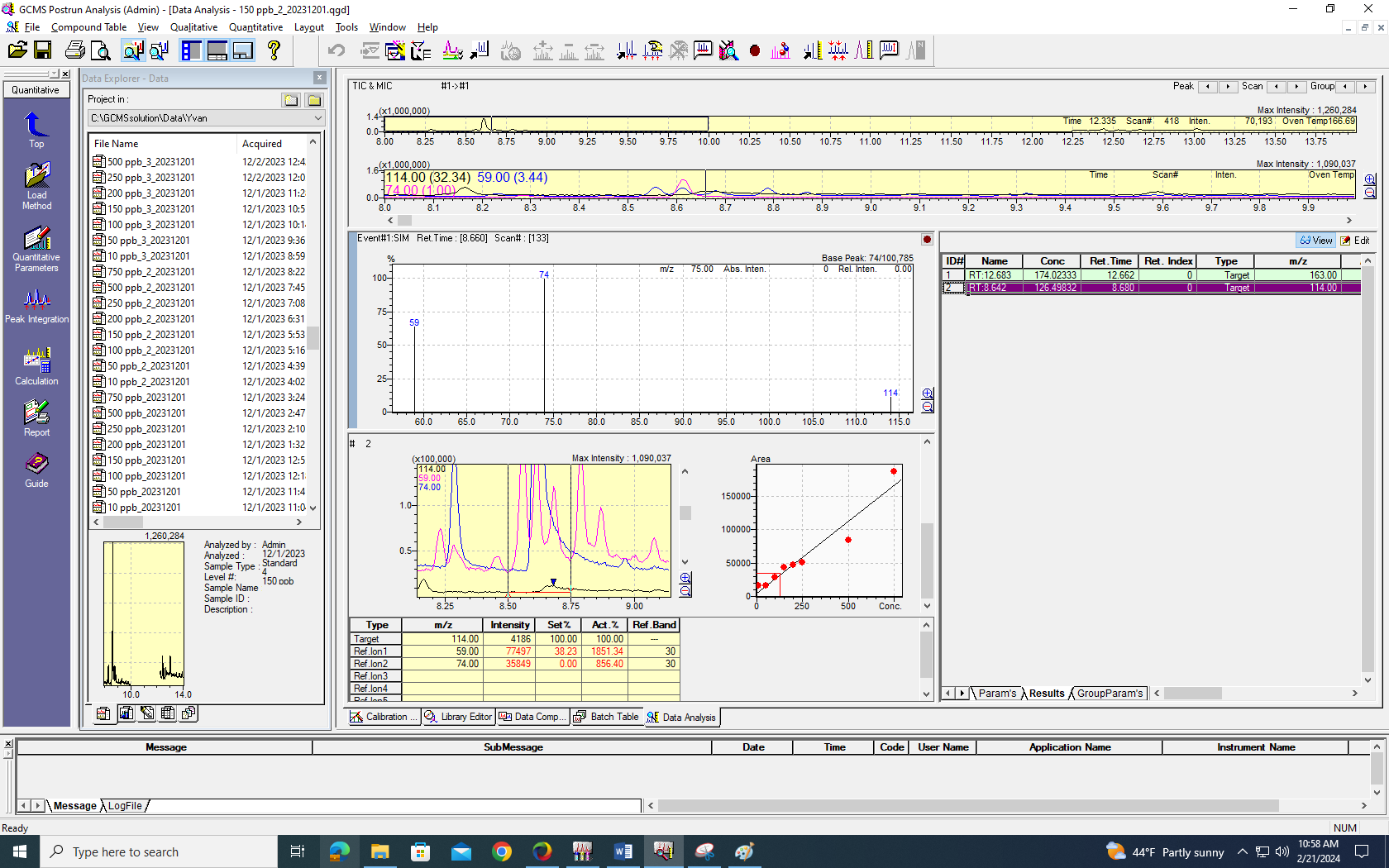

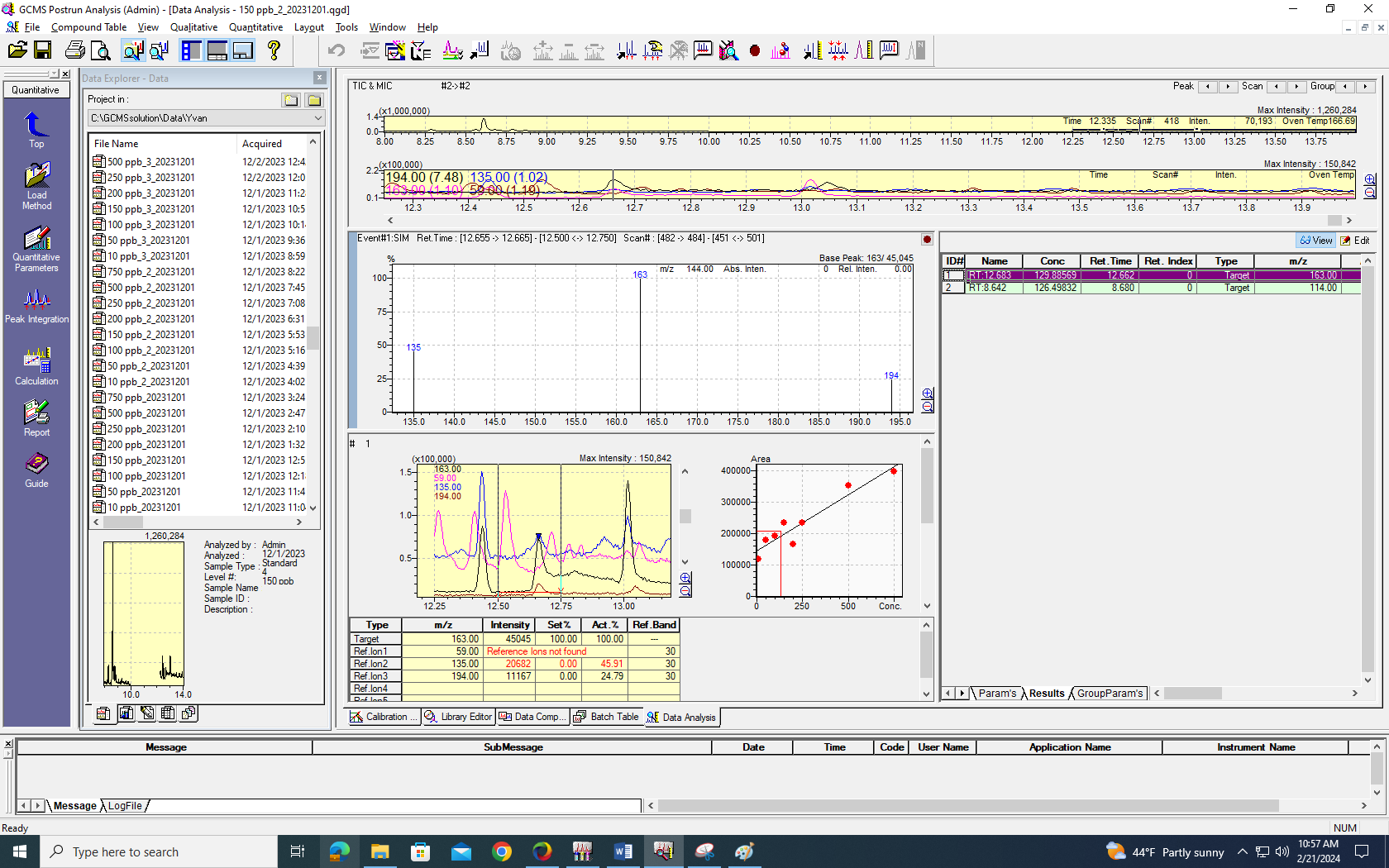


Adipic Acid, dimethyl ester (RT: 8.6 min), Manual integration for ion 114 m/z between 8.5 min and 8.75 min

Terephthalic acid, dimethyl ester (RT: 12.6 min), Manual integration for ion 163 m/z between 12.5 min and 12.75 min


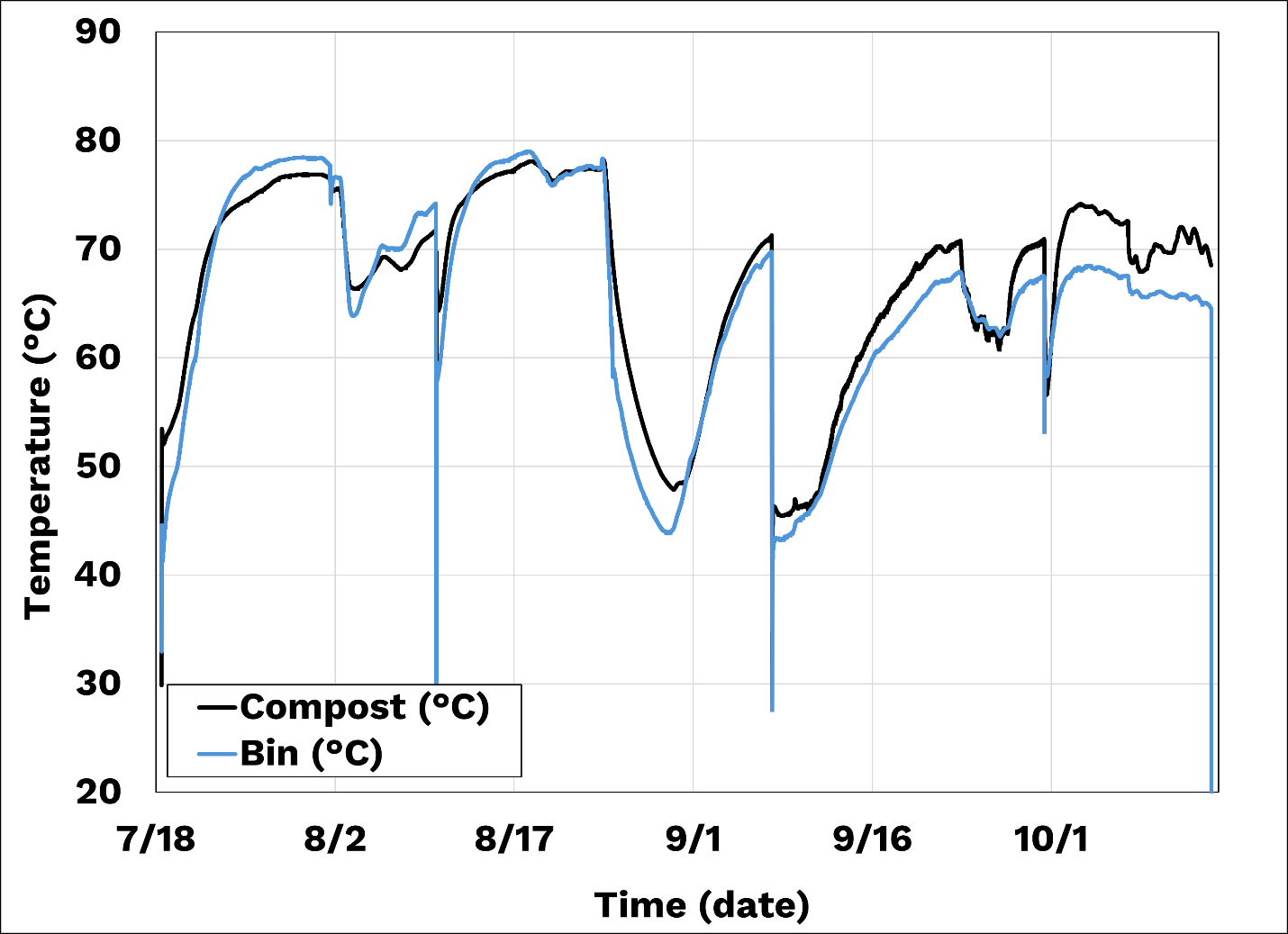


*Figure S2. Industrial compost temperatures.*


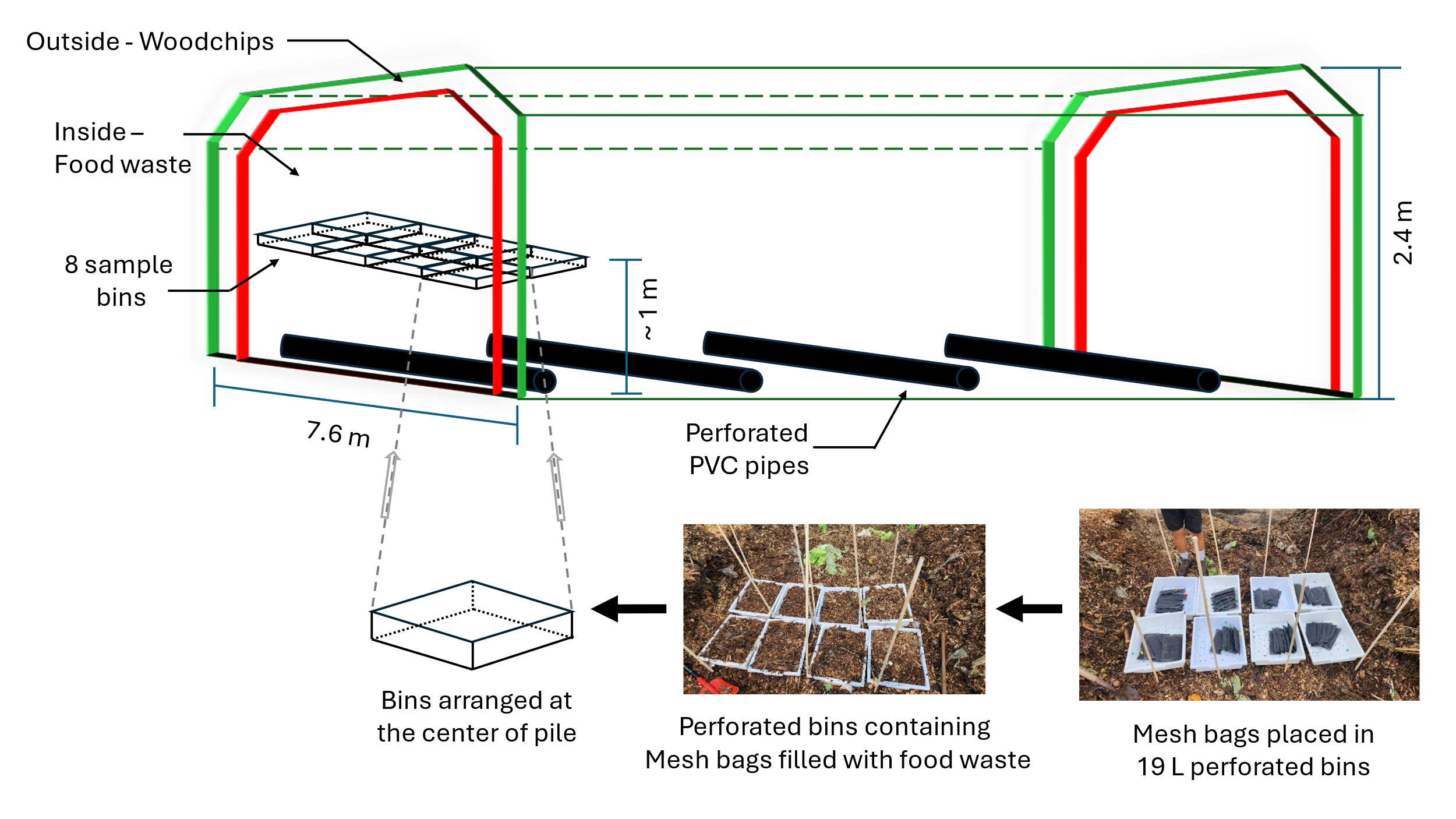


Figure S3. Industrial compost schematic and sample location

For the industrial compost represented in Figure S3, bins were made from polypropylene. The bins have been previously tested extensively at room temperature for extended periods using soil burial techniques. The bins do undergo weathering but not biodegradation. Regarding the soil into the meshbags, 100 grams of sieved "Bovung manure mix" (<5 mm) was placed inside mesh bags on each side of the plastic film, sandwiching it between 200 grams of soil. This was done for all samples to ensure consistent film-to-compost contact. The small soil particles helped create uniform contact across the surface of the films, ensuring uniform degradation and prevented direct disintegration of the plastic into the IC, allowing for easier retrieval of plastic fragments mixed with the soil for further analysis. Finally, the outer soil layers protect the plastic from sticking to the mesh bags at higher temperatures. samples were too degraded to clean. They were directly dried overnight and weighed. As Figure S3 shows we had ~6 mesh bags per bin and eight 5-gal bins total.

**
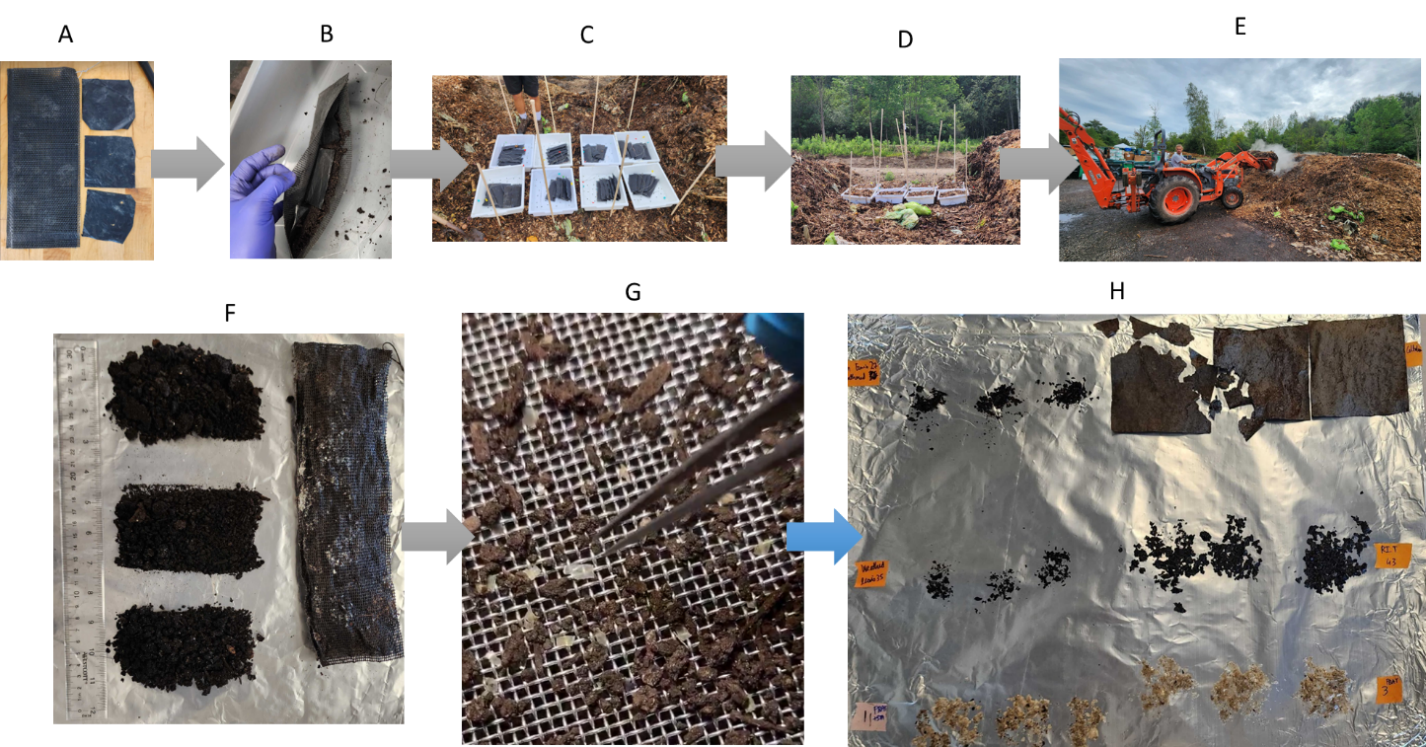
** Figure S4. Industrial compost sampling procedure

Figure S4 details the sampling procedure. Briefly, triplicate PBAT film samples were prepared (A). A total of eight mesh bags were prepared. 100 grams of sieved soil (<5 mm) was added inside the mesh on each side of the triplicate films (B) as described previously figure S3 (D). PBAT samples were sandwiched between soil because the mesh opening was small (~1 mm^2^) and particle size of the unground compost material being large and uneven. Which made it difficult to ensure uniform “film to compost” contact ratio (i.e. film surface area that is in contact to compost) for all samples equally. Adding small particle size soil to the mesh bags elevates the non-uniform surface area issue. So as not to lose the sample bags into the large pile (dimensions unknown, height ~ 10ft to 12ft), bags were placed in eight identical 5-gallon bins (C). Each bin represented one time point for sampling. Bins are now filled with composting material and mesh bags are then placed into the bins about 2 to 3 cm apart from each other. After which the bins are placed in the center of the pile (~ 4ft from the from the ground) (D). Finally, the bins are covered with about 4’ to 5’ composting material (E).

At regular intervals, samples were retrieved by carefully digging the pile from one side and pulling the bins out. Mesh bags were carefully retrieved from the bins are stored in Ziplock bags to be transported back to the lab. Mesh bags are opened over a clean aluminum foil and soil pulled out. If the samples were too fragmented, the soil was divided approximately into three parts (F). Triplicate soil is then dried at 60°C for 24 hours. The soil is then passe through size 14 mesh, plastic pieces over the mesh are retrieved and weighed (G). A picture of the fragmented samples can be seen in (H).
